# Reducing the Temperature

**Published:** 1867-07

**Authors:** 


### *Reducing the Temperature.*

At the late meeting of the Illinois State Medical Society we regretted to hear, after the triumphant demonstration by the sphygmograph, that three fingers of Bourbon whiskey produced the same gyrations of the pulse wave and reduction of animal heat as three weeks of typhoid fever, several irreverent M.D.'s simultaneously exclaim: "It's awfully hot—let's go and *reduce the temperature*," which they did, literally, "in a horn."

